# Clinical and immunological allergy assessment in cancer patients

**DOI:** 10.1038/s41598-021-97200-y

**Published:** 2021-09-13

**Authors:** Bruno Gustavo Muzzi Carvalho Carneiro, Andy Petroianu, José Augusto Nogueira Machado, Paula Martins Ferreira dos Anjos, Fabiana Rocha da Silva, Luiz Ronaldo Alberti, Vivian Resende, Sofia Candia Barrientos

**Affiliations:** 1 Surgery Department of the School of Medicine of the Federal University of Minas Gerais, Belo Horizonte, Brazil; 2 Service of Oncology of the Alberto Cavalcanti Hospital of the Hospital Foundation of the State of Minas Gerais, Rua Rio Claro 235, ap 401, Belo Horizonte, MG 30411-235 Brazil; 3 Santa Casa Hospital of Belo Horizonte, Belo Horizonte, Brazil

**Keywords:** Cancer epidemiology, Cancer microenvironment, Cytokines

## Abstract

Cancer is associated with immunodeficiency, while allergies result from immune system hyperactivity mediated by cytokines and immunoglobulins. The purpose of this study was to determine the relationship between immune environment of specific cancers and allergies, emphasizing cytokines related to Th1 and Th2 responses associated with IgE. 80 adults were distributed into two groups: control (n = 20) and cancer (n = 60), distributed in three subgroups (n = 20), head and neck, stomach, and prostate cancers. This study compared Th1 (IL-2) and Th2 (IL-4) parameters, anti-inflammatory, pro-inflammatory, or regulatory profile regarding both IgE levels and reported allergies, by means of clinical manifestations and IgE, IL-1β, IL-2, IL-4, IL-17, and TGF-β serum concentration. Clinically allergies were observed in 50% of the control group and in 20% of the cancer group (*p* = 0.009). IL-2 cytokine and TGF-β concentrations were higher in the patients with cancer as compared to the control (*p* < 0.005). However, there were IL-4, IL-17, and IL-1β decreases in the patients with cancer (*p* < 0.05). No correlation was observed between the cytokines studied and IgE and clinically proven allergies in both investigated groups. There was an inverse association between cancer and clinical allergy manifestations. In head and neck, stomach, and prostate cancers, an immunosuppressive serum tumor environment was predominant. There was no difference in cytokines related to Th1 and Th2 parameters in relation to IgE. No correlation was found between clinically proved allergies and immunity markers related to the same allergens.

## Introduction

The relation between cancer and allergies may be connected to immunodeficiency and immune surveillance, triggered by each disease^1,2^. Allergic reactions result from the hyperactivity of the (Th)2 auxiliary T-immune response, which activates eosinophils, macrophages, and IgE, by means of IL-4, IL-5, and IL-13 secretion^3,4^. As a result, immune surveillance and cell damage, including malignant cells, are increased^1,3,5^. The IL-4 increase may direct the antibody production to the IgE isotype, associated with type I hypersensitivity reactions. This allergy process, mediated by IgE, is capable of inhibiting tumor growth due to its cytotoxic effect on cancer cells^6–11^.

According to the immune surveillance concept, the immune system destroys neoplastic cells by means of innate immunity, responsible for immediate detection and destruction of malignant cells^12,13^. In primary or acquired immunodeficiency, as in acquired immunodeficiency syndrome, when immunosuppressants are used, and in elderly patients, there is a predisposition or a higher probability for the occurrence of cancer^14–17^.

Allergo-oncology is a recent multidisciplinary area that studies possible associations between cancer and the Th2 subdivision in the immune system, as well as the relations between tumors and immunity mediated by IgE and cytokines, to develop immunotherapies for cancer control^1,9,18,19^. Immune mechanisms found in allergic processes indicate new immunotherapy and immunoprophylaxis for this disease^20^.

Th1 cells offer protection against intracellular pathogens, contributed to the occurrence of autoimmune and chronic inflammatory diseases, as well as secreting cytokines, such as IL-2, IL-12, tumor necrosis factor-β (TNF-β), and interferon-γ (IFN-γ)^4,21^. Other lymphocyte populations have been described in the literature, especially for Th17, which secretes IL-17 associated with autoimmune and extracellular defense diseases, as well as for regulatory T cells (Treg), associated with immune tolerance, inhibiting effector T cells (Tef), and suppressing destruction by autoantigens^4,21–26^.

IL-1β induces a powerful inflammatory response and plays a critical role in the organism’s fight against pathogens and harmful agents. However, excessive activation or failure in interrupting the inflammatory response may lead to cell or tissue lesions, resulting in allergy processes, chronic autoimmune inflammatory diseases, and cancer^27,28^. The transforming growth factor-β (TGF-β) plays a two-phase role in cancer development and advances: in normal cells, TGF-β suppresses tumor genesis. However, once the tumor process has already begun, TGF-β creates an immunosuppressive microenvironment by inhibiting IL-2, IL-12, and IFN-γ. Therefore, local immune tolerance is created, and the TGF-β serum concentration is increased, which is frequent in cancer patients^29–32^.

The allergy process is assessed by clinical assessment, skin tests, and total or specific IgE serum levels. However, no direct relation between specific reported allergies nor skin sensitivity to the same allergen has been observed, understanding that a clinical allergy may be accompanied by a negative skin test for the same sensitivity agent^33^. Positive skin tests are observed in people who do not exhibit clinical allergies. In the presence of cancer, a lower rate of reported allergies was observed for the general population, even with a higher incidence of positive skin tests for allergens^33^. The IgE serum measurement (type I hypersensitivity reaction marker) has been used as an objective diagnostic method for allergies. The inverse association between IgE concentration and cancer may be specific to the allergy and tumor types^6,20,34,35^.

The mechanism proposed to explain the association between cancer and allergy is that in allergy a heightened immune response is prevalent, whereas in cancer a hyporesponsive immune response is observed. Thus, in allergic people there would be an increase in production of cytokines related to the Th2 response and increasing the production of IgE against the specific tumor antigen, capable of activating effector cells and destroying tumors^36–39^. However, few studies investigate the measurement of cytokine and other biomarkers in this situation^36,37^. Almost all trials involving cytokines are conducted in animal models^19^, and only two studies with human subjects have been identified^38,39^.

The purpose of this study, which is part of a line of research^33,40,41^, was to determine the relationship between immune environment of specific cancers and allergies, emphasizing cytokines related to Th1 and Th2 responses associated with IgE.

## Results

Demographic data are described in Table 1. There was a predominance of male subjects in the cancer patient group, and the prevalence of alcoholism and smoke was higher in the cancer group. The average age of the participants was 63 (± 10) years and there was no difference between the groups (*p* > 0.05). Cancer staging was I—3%, II—24%, III—38%, and IV—35%.

**Table 1:** Characterization of the studied sample.

Characteristics		Groups—N (%)		
		Control	Cancer	*p*
Gender	Female	4 (20.0)	8 (13.3)	0.470
	Male	16 (80.0)	52 (86.7)	
Age bracket	Under 63 years of age	13 (65.0)	27 (45.0)	0.121
	Over 63 years of age	7 (35.0)	33 (55.0)	
Comorbidities	Absent	9 (45.0)	28 (46.7)	0.897
	Hypertension	6 (30.0)	15 (25.0)	
	Diabetes	0 (0)	3 (5.0)	
	Dyslipidemia	0 (0)	1 (1.7)	
	2 types of chronic disease	2 (10.0)	7 (11.6)	
	> 3 types of chronic disease	2 (10.0)	5 (8.3)	
	Migraine	1 (5.0)	1 (1.7)	
Alcoholism	Absent	14 (70.0)	32 (53.3)	0.010
	Social	5 (25.0)	4 (6.7)	
	Daily	0 (0)	11 (18.3)	
	Former alcoholic	1 (5.0)	13 (21.7)	
Smoking	Never	15 (75.0)	27 (45.0)	0.020
	Former smoker	1 (5.0)	22 (36.7)	
	Currently smoker	4 (20.0)	11 (18.3)	
Allergy	Absent	10 (50.0)	48 (80.0)	0.009
	Present	10 (50.0)	12 (20.0)	
Type of allergy	Absent	10 (50.0)	48 (80.0)	0.005
	Allergic rhinitis	7 (35.0)	9 (15.0)	
	Medication	2 (10.0)	0 (0)	
	Food	0 (0)	3 (5.0)	
	Atopic dermatitis	1 (5.0)	0 (0)	
	Asthma	0 (0)	0 (0)	

Pearson's chi-squared test. N = 80.

Allergies were reported in 50% of the control group and in only 20% of the cancer group (OR 0.46, 95% CI 0.26–0.81, *p* = 0.009). There was an inverse relation in the comparison between the control group and the prostate adenocarcinoma group (OR 0.17, 95% CI 0.04–0.79, *p* = 0.024). However, no difference was observed in the comparison with the gastric adenocarcinoma group (OR 0.25, 95% CI 0.06–1.02, *p* = 0.053), or with the head and neck squamous cell carcinoma (HNSCC) group (OR 0.33, 95% CI 0.09–1.27, *p* = 0.108). The most common type of allergy was rhinitis, which was observed in 15% of the cancer patients and in 35% of the control individuals (*p* = 0.005) (Table 1).

The agreement between reported allergies and positive IgE (considered to be an IgE concentration ≥ 25 UI/ml) was weak in all groups: κ = 0.000 in the control group, κ = 0.002 in the cancer patient group, namely κ = −0.037 for prostate adenocarcinoma, κ = 0.237 for gastric adenocarcinoma, and κ = −0.250 for HNSCC.

An IL-2 increase was observed in HNSCC patients in relation to the control (*p* = 0.005). In the cancer patient group, there was a reduction in IL-4 (prostate: *p* = 0.001, HNSCC: *p* = 0.045, gastric: *p* = 0.046), IL-17 (prostate: *p* = 0.034, HNSCC: *p* = 0.001, gastric: *p* = 0.004), and IL-1β (prostate: *p* = 0.012, gastric: *p* = 0.049), and an increase in TGF-β for this group (prostate: *p* = 0.001 and gastric: *p* = 0.001). No relation was observed between cytokines and cancer staging. (Table 2).

**Table 2:** Relationship among cancer, reported allergies, IgE (UI/ml), and cytokine (pg/ml) concentration.

	Characteristic	IgE		IL-2		IL-4		IL-17		IL-1β		TGF-β	
		Median	*p*	Median	*p*	Median	*p*	Median	*p*	Median	p	Median	p
Group	Control	192.7	0.158	52.0	0.139	31.9	0.005	573.9	0.001	7.3	0.060	495.9	0.000
	Cancer	93.7		66.3		23.6		373.7		5.3		969.8	
Reported allergies	Absent	108.8	0.690	58.6	0.561	28.7	0.119	404.2	0.075	5.4	0.772	737.1	0.605
	Present	54.1		58.0		31.1		540.0		5.5		750.0	
Tumor type	Control	192.7	–	52.0	–	31.9	–	573.9	–	7.3	–	495.9	–
	Prostate	96.5	0.191	52.7	0.837	12.4	0.001	492.4	0.034	4.2	0.012	1193.5	0.001
	HNSCC	188.6	0.766	86.7	0.005	25.0	0.045	235.5	0.001	6.5	0.635	659.5	0.071
	Stomach	34.3	0.065	58.1	0.511	26.7	0.046	404.2	0.004	5.3	0.049	1107.0	0.001
Staging General Cancer	Control	192.7	–	52.0	–	31.9	–	573.9	–	7.3	–	495.9	–
	I	12.1	0.045	74.7	0.265	31.2	0.974	319.8	0.053	9.2	0.839	722.7	0.819
	II	51.9	0.052	46.0	0.773	25.1	0.043	436.1	0.029	4.5	0.041	926.7	0.019
	III	77.3	0.244	69.7	0.113	25.5	0.008	420.4	0.002	5.0	0.029	1301.5	0.000
	IV	188.6	0.555	86.7	0.092	20.4	0.014	286.0	0.001	5.6	0.319	810.4	0.026

Kruskal–Wallis test. Comparisons in relation to the Control Group, or to the Reported Allergies Absent Subgroup. Values are presented as median. Groups: Control (n = 20) and General Cancer (n = 60), that was the sum of three subgroups: Prostate (n = 20), HNSCC (n = 20), and Stomach (n = 20) Cancer. Reported allergies absent (n = 48) and present (n = 22). Staging General Cancer I (n = 3), II (n = 14), III (n = 23), IV (n = 20).

Abbreviations: IgE = immunoglobulin E, IL = interleukin, TGF = transforming growth factor, HNSCC = head and neck squamous cell carcinoma.

Staging based on *AJCC Cancer Staging Manual, 8th ed.*^43^*.*

**Figure 1:**
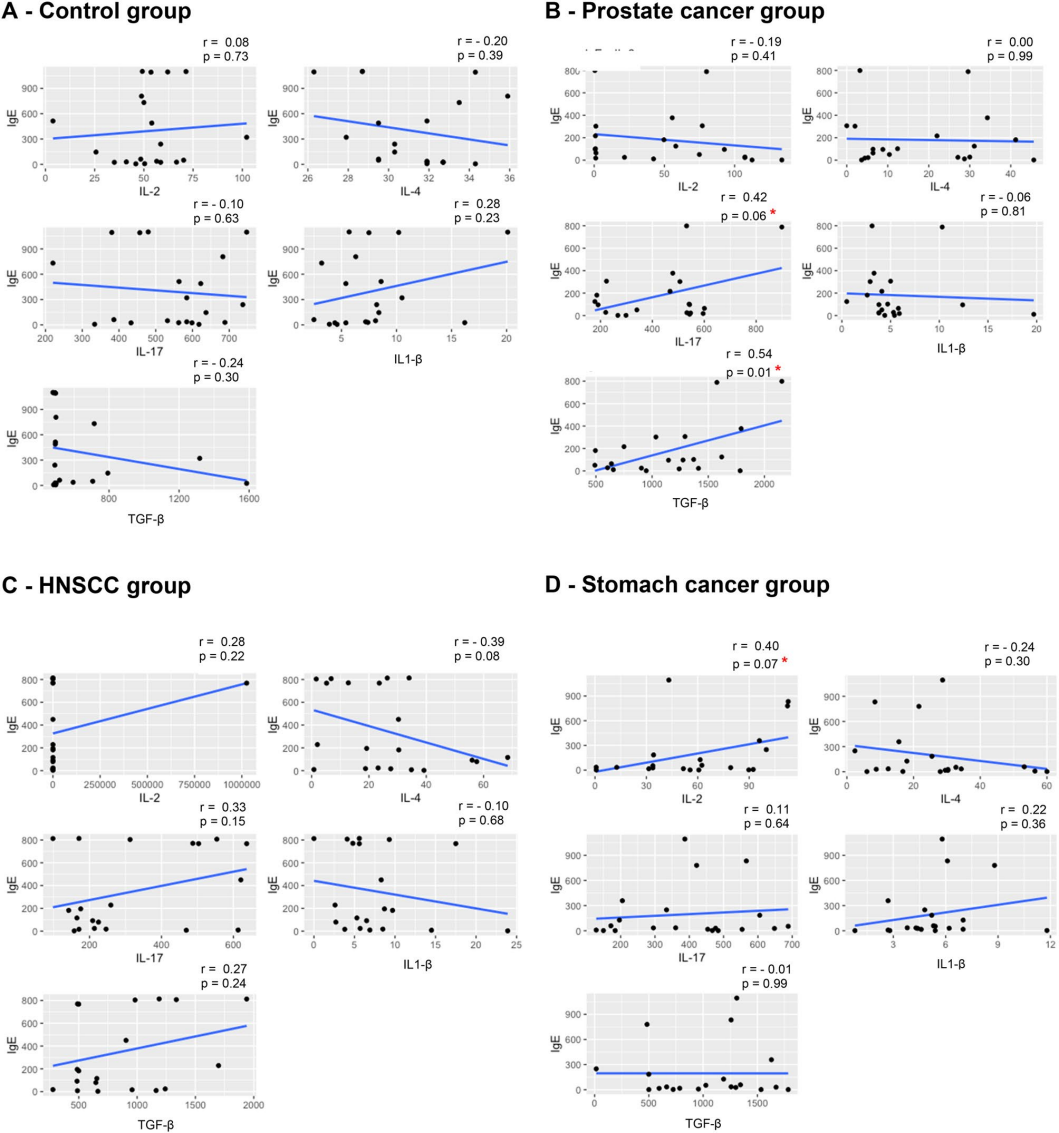
Dispersion diagrams and linear regression axes based on the correlation factors between cytokines (pg/ml) and IgE (UI/ml), stratified by patient group. Spearman Correlation Test. * Groups with a moderate correlation (*Devore JL *^45^*)*. Groups: Control (n = 20), Prostate cancer (n = 20), HNSCC (n = 20), Stomach cancer (n = 20). Abbreviations: IgE = immunoglobulin E, IL = interleukin, TGF = transforming growth factor, HNSCC = head and neck squamous cell carcinoma.

There was no difference in the values of cytokine medians between the two groups, based on IgE concentrations or on the classification of IgE in the normal, borderline, or high categories (p > 0.05) (Table 3). There was a weak correlation between most cytokines and IgE levels in both groups (Spearman’s correlation factors < 0.4 for most groups)^45^. A moderate correlation was identified only in the prostate adenocarcinoma group for TGF-β (r = 0.54, *p* = 0.01) and IL-17 (r = 0.42, *p* = 0.06) as well as for gastric adenocarcinoma (r = 0.40, *p* = 0.07) for IL-2 (Fig. 1). There was no correlation between IgE concentrations and staging in the general cancer group (r = 0.25, *p* = 0.60), prostate cancer (r = 0.12, *p* = 0.62), HNSCC (r = 0.07, *p* = 0.78), and stomach cancer (r = 0.22, *p* = 0.37).

**Table 3:** Cytokine (pg/ml) values among groups and IgE (UI/ml) concentrations.

Group	Cytokines	IgE concentration classification			*p*
		Normal (< 25 UI/ml)	Borderline (25–100 UI/ml)	High (> 100 UI/ml)	
Control	IL-2	48.15 (35.00–58.48)	56.45 (41.07–70.22)	53.62 (3.82–102.20)	0.651
	IL-4	32.69 (31.89–34.29)	31.89 (29.48–32.69)	30.28 (26.28–35.89)	0.221
	IL-17	515.73 (333.65–617.73)	562.44 (386.08–688.28)	584.37 (220.21–747.38)	0.691
	IL1-β	4.51 (3.93–5.40)	7.38 (2.51–16.21)	8.19 (3.22–20.06)	0.074
	TGF-β	496.35 (483.30–1,586.00)	517.20 (491.90–706.80)	493.60 (479.50–1,318.00)	0.611
General cancer	IL-2	62.63 (0.74–203.69)	48.11 (0.67–107.34)	78.51 (0.49–1,025,523.40)	0.329
	IL-4	27.80 (0.80–60.00)	28.54 (6.40–57.60)	21.60 (1.60–68.40)	0.465
	IL-17	376.07 (128.69–612.97)	350.33 (171.59–688.28)	404.19 (98.19–898.95)	0.776
	IL1-β	5.55 (0.83–23.92)	4.89 (2.71–12.40)	5.20 (0.53–17.48)	0.611
	TGF-β	952.55 (278.40–1,784.00)	965.05 (485.20–1,672.00)	1,111.50 (14.70–2,155.00)	0.866
Prostate Cancer	IL-2	107.30 (1.08–133.62)	48.25 (0.67–107.34)	49.67 (0.49–79.97)	0.242
	IL-4	5.20 (3.60–45.60)	9.60 (6.40–30.00)	25.80 (2.00–41.20)	0.743
	IL-17	543.38 (266.92–595.81)	436.61 (190.66–600.57)	478.55 (178.27–898.95)	0.594
	IL1-β	5.45 (3.77–19.71)	4.71 (3.77–12.40)	3.27 (0.53–10.27)	0.108
	TGF-β	1,241.00 (655.10–1,784.00)	771.50 (490.60–1,275.00)	1,370.00 (495.20–2,155.00)	0.091
HNSCC	IL-2	68.65 (1.27–203.69)	81.43 (70.84–92.01)	96.57 (0.59–1,025,523.40)	0.383
	IL-4	25.40 (0.80–39.20)	56.80 (56.00–57.60)	21.40 (1.60–68.40)	0.123
	IL-17	229.74 (157.29–612.97)	216.87 (208.77–224.98)	285.99 (98.19–636.80)	0.891
	IL1-β	7.71 (3.83–23.92)	4.61 (2.71–6.52)	5.60 (2.56–17.48)	0.404
	TGF-β	810.35 (278.40–1,241.00)	565.85 (485.20–646.50)	780.10 (486.60–1,939.00)	0.409
Stomach Cancer	IL-2	55.51 (0.74–92.37)	33.78 (0.69–79.24)	96.02 (34.19–112.80)	0.068
	IL-4	29.48 (6.00–60.00)	31.49 (8.80–53.20)	18.00 (2.40–28.68)	0.152
	IL-17	453.77 (128.69–553.86)	418.49 (171.59–688.28)	387.04 (196.38–605.34)	0.655
	IL1-β	4.64 (0.83–11.84)	4.89 (3.77–6.16)	5.75 (2.66–8.80)	0.560
	TGF-β	784.40 (500.00–1784.00)	1,279.50 (663.70–1672.00)	1,189.00 (14.70–1629.00)	0.462

Kruskal–Wallis Test. Values are presented as median (min–max).

Groups: Control (n = 20) and General Cancer (n = 60), that was the sum of three subgroups: Prostate (n = 20), HNSCC (n = 20), and Stomach (n = 20) Cancer.

Abbreviations: IgE = immunoglobulin E, IL = interleukin, TGF = transforming growth factor, HNSCC = head and neck squamous cell carcinoma.

Classification of IgE Levels based on *Wiemels JL *et al*.* ^44^.

To determine whether the presence of reported allergies is associated with the predominance of some immunity markers, cytokines (Th1 (IL-2), Th2 (IL-4), Th17 (IL-17), IL-1β, TGF-β) and IgE were compared between both groups. Table 4 shows there was no difference among these variables in this study.

**Table 4:** Cytokine (pg/ml) and IgE (UI/ml) values among the groups and reported allergies.

Group	Cytokines and IgE	Reported allergies		*p*
		Absent	Present	
Control	IL-2	55.24	47.34	0.218
	IL-4	29.88	32.29	0.105
	IL-17	456.15	591.52	0.190
	IL1-β	5.55	7.81	0.218
	TGF-β	491.75	496.50	0.075
	IgE	364.30	97.40	0.393
General cancer	IL-2	64.637	67.739	0.956
	IL-4	23.6	24.337	0.763
	IL-17	363.68	414.681	0.890
	IL1-β	5.449	3.952	0.191
	TGF-β	956.9	1249.5	0.327
	IgE	99.65	76.90	0.671
Prostate cancer	IL-2	55.64	21.64	0.616
	IL-4	17.20	5.20	0.254
	IL-17	468.06	532.89	0.305
	IL1-β	4.69	3.77	0.146
	TGF-β	1146.00	1413.00	0.305
	IgE	97.39	85.20	0.731
HNSCC	IL-2	87.63	72.67	0.866
	IL-4	23.60	34.80	0.266
	IL-17	245.95	224.98	0.965
	IL1-β	6.72	5.65	0.964
	TGF-β	655.10	663.80	0.612
	IgE	228.55	203.80	0.119
Stomach cancer	IL-2	58.07	48.32	0.750
	IL-4	28.34	20.54	0.820
	IL-17	437.56	282.65	0.617
	IL1-β	5.42	4.48	0.211
	TGF-β	990.95	1301.00	0.494
	IgE	117.47	121.65	0.211

Kruskal–Wallis Test. Groups: Control (n = 20) and General Cancer (n = 60), that was the sum of three subgroups: Prostate (n = 20), HNSCC (n = 20), and Stomach (n = 20) Cancer.

Abbreviations: IgE = immunoglobulin E, IL = interleukin, TGF = transforming growth factor, HNSCC = head and neck squamous cell carcinoma.

## Discussion

The relation between cancer and allergies has been studied since the 1950s^46^, and has yielded conflicting results, due to the diversity of study methods and to risk factors for cancer or allergies, such as age, use of medication and associated diseases, often not controlled^6,38,39^. These variables were observed and carefully adjusted in this study, to avoid interfering in the interpretation of results and to preserve their reliability.

There was an inverse association between cancer and clinically detected allergies, which favors the theory of immune surveillance^12,47^, which is higher in allergic processes and may be associated with the damage on malignant cells^1,5^. In this study, as well as in other investigations conducted in the same line of research, there was a lower prevalence of allergies in cancer patients, especially in the presence of prostate adenocarcinoma, in comparison with the control group^33,40^. There was also an inverse association between reported allergies and the presence of HNSCC, as well as of gastric adenocarcinoma^34,37,48,49^. The lack of significance in this comparison in the present study, which was revealed only as a trend, is probably due to the small number of cases, although we have studied a larger number of individuals than has been described in the literature in similar studies^38,39^.

A pivotal limitation of this work was the low number of participants per group due to the specificity of cancers, which were investigated. The rigorous statistical analysis of all parameters leads this study suitable to drive conclusions concerning the relationship between allergies and cancer. Other types of cancer could be included in this study, which belong to future surveys of the same line of research. These upcoming studies will expand the current data with more participants.

Allergy is a complex disorder that involves immune factors that may or may not depend on IgE, and few studies are available that evaluate the IgE serum dosage in cancer patients^35,50–52^. Although IgE is useful in detecting allergies, this immunoglobulin is not increased in all types of allergic reactions, which may explain the low concordance between reported allergies and positive IgE observed in this work, likewise as was reported in another study^44^.

When the inverse association between cancer and allergies is detected, immune surveillance and immunoprophylaxis theories are mentioned as an explanation. Immune surveillance theory suggests that reactions to allergens increase the immune response, including the production of IgE and the activation of effector immune cells (monocytes, macrophages, *natural killer* cells, eosinophils, mastocytes, and basophils), capable of detecting and destroying cancer cells. Activation of the Th2 response observed in allergic conditions promote the synthesis of related cytokines, such as IL-4 and IL-3, which favors the synthesis of IgE against specific tumor antigens, capable of activating effector cells and destroying tumors^19,36,37^. Immunoprophylaxis theory is related to inhaled antigens and allergic reaction manifestations (coughing, sneezing, etc.) intended to eliminate these antigens by means of IgE and activated effector cells that damage and remove infectious microorganisms, mutagenic toxins, and environmental carcinogens^34,36,37^.

Only two studies evaluated the measurement of cytokines and other biomarkers, connecting cancer and allergies^38,39^. In a study comparing cytokines from HNSCC to the allergic rhinitis group and the control group (with no cancer or allergies), no difference was observed among the concentrations of the studied cytokines (response Th1 (IL-2, IL-12, IFN-γ, TNF-α), Th2 (IL-4, IL5, IL-13), innate immunity (IL-1β, IL-8, IL-17), innate immunity (MCP)-1, macrophage inflammatory protein (MIP)-1β, granulocyte and macrophage colony stimulating factor (G-CSF), granulocyte and macrophage colony stimulating factor (GM-CSF), and immunity related to the T cells (IL-6, IL-7, IL-10)^38^. A different study with breast cancer patients revealed no differences in cytokine concentrations (IL-1β, IL-6, IFN-γ, IL-4) between the cancer and allergy groups^39^.

The fact that groups were not paired by age, which may have interfered in the results, as the immune response changes with age, and that a lower number of patients, if compared to the present work, was involved, are limitations in these two previous studies^38,39^. Results in this study, as well as in those mentioned in the literature, indicated no difference in cytokines related to Th1 and Th2 parameters in the association between cancer and allergic processes related to type I hypersensitivity reactions. This finding contradicts one of the proposed theories in epidemiological study literature about cancer and allergy, in which a difference in cytokines related to Th1 and Th2 parameters had been suggested^19,36,37^.

New studies have increased the knowledge beyond the immune system’s Th1/Th2 paradigm and other subsets of cells involved in this process, such as Th3, Th17, Th9, follicular Th, and regulatory T, illustrating that these interrelations are broader. An additional hypothesis, other than the Th1/Th2 paradigm, is based on publications arguing that T CD4+ lymphocytes exhibit plasticity and do not behave as inflexible Th1 or Th2, producing only a few cytokines^53,54^, despites being flexible in the synthesis of mediators and in the conversion of effector cells Th1, Th2, Th17, or regulatory T^21–23,55^. Based on the action of certain cytokines, a subset of T cells may be transformed into different cytokine secretory cells^21–23,55,56^.

Immune modulating cytokines (TGF-β, IL-10, IL-17, and IL-23) facilitate tumor development, while others (IFN-γ, TNF-α, GM-CSF, IL-2, IL-6, IL-17, and IL-12) promote immune surveillance and delay its growth^19,57^. Studies discussing cancer and allergies must evaluate these literature findings.

To determine whether there is a correlation of other mediators potentially involved in the relation between cancer and allergies, other cytokines were studied. An increase in regulatory cytokine IL-2 and TGF-β was detected, as was the reduction of IL-4, IL-17, and IL-1β, reflecting the cancer patient's immunosuppressive environment and the lower prevalence of allergies. Such findings and characteristic cytokine levels in tumors confirm the specificity of the immune response according to the type of cancer^32^. The moderate correlation between IgE and IL-17 and TGF-β in the prostate adenocarcinoma group suggests the presence of an allergy marker with immunosuppressive response and immune response specificity, according to the tumor type. Studies show that TGF-β does not have a direct effect on the origin of the Th17 cells; however, it may favor its development to suppress the Th1 differentiation^58,59^.

A better understanding of the mechanisms involved in the relationship between cancer and allergies require studies involving a higher number of cytokines and their relation with the molecular messenger (messenger RNA) responsible for synthesizing the cytokines involved in allergies and inflammation^23,60^. Future studies should involve the evaluation, in control environments, of the immune system to exogenous antigens that trigger allergies in cancer patients, in a dynamic study of antigen stimulus and its response in the immune system^61–63^.

## Conclusions

There was an inverse association between cancer and clinical allergy manifestations. In head and neck, stomach, and prostate cancers, an immunosuppressive serum tumor environment was predominant. There was no difference in cytokines related to Th1 and Th2 parameters in relation to IgE. No correlation was found between clinically proved allergies and immunity markers related to the same allergens.

## Methods

### Ethics approval and consent to participate

This research was carried out according to the principles set out in the Declaration of Helsinki 1964 and all subsequent revisions. This work was approved by the Research Ethics Committee of the Hospital Foundation of the State of Minas Gerais, under protocol number 075/2009. Informed consent was obtained from all participants.

### Patient recruitment, characteristics and sample collection

This control case research evaluated the cytokines related to the Th1 (IL-2) and Th2 (IL-4) parameters, in relation to the IgE levels, and to reported allergies in control individuals and cancer patients. The levels of immune mediators involved in the Th17 (IL-17) response, in both immunosuppression/immunoregulation (TGF-β) and IL1-β pro-inflammation in relation to IgE levels and to reported allergies in control individuals and cancer patients were also evaluated.

This study included patients with a recent histological diagnosis of cancer and they were only admitted to the research before undergoing any type of treatment. The individuals included were older than 18 years of age, from both genders, who presented an *Eastern Cooperative Oncology Grou*p (ECOG) performance score between zero and two^42^, TNM staging from I to IV^43^, and no previous history of other cancer, autoimmune disease or immune treatment, corticosteroid therapy, chemotherapy, or radiation therapy.

The control group consisted of 20 volunteers, with no cancer and using no medications, selected by age to be paired with the cancer group. The clinical assessment was conducted to identify addictions, alcoholism and smoking, associated diseases, medications, allergy history, allergy type (allergic rhinitis, atopic dermatitis, asthma, food and drug allergies), as well as allergic symptoms after exposure to certain allergens, such as medication, food, dust, mold, or animals. Information related to allergy was specifically asked to all patients and volunteers, emphasizing details related to each allergic manifestation. All reported allergies were proved by medical diagnosis, identifying allergens for each case.

The present study included 60 cancer patients, distributed in three subgroups (n = 20), gastric adenocarcinoma, prostate adenocarcinoma, and head and neck squamous cell carcinoma (HNSCC). These tumors were selected because they exhibited an inverse relation with allergies in a previous epidemiological study, within the same line of research^40^. All patients in both groups were identified by gender and age. Cancer patients were subjected to the same clinical assessment as the control group, regarding allergies and their relation to cancer, considering its histology, location, staging, and diagnostic data.

Serum cytokine dosages were obtained using the ELISA method, in accordance with manufacturer instructions. This study reviewed total IgE (ALPCO, Salem, NH, USA), IL-2 (Enzo Life Sciences, Farmingdale, NY, USA), IL-4 (Enzo Life Sciences, Farmingdale, NY, USA), TGF-β (Xpressbio Life Science, Frederick, MD, USA), IL1-β (Xpressbio Life Science, Frederick, MD, USA), and IL-17 (Xpressbio Life Science, Frederick, MD, USA).

### Statistical analysis

Statistical analyses were conducted using the *Statistical Package for the Social Sciences* software, version 25 [https://www.ibm.com/support/pages/downloading-ibm-spss-statistics-25]^44^. The Kolmogorov–Smirnov normality test was conducted to assess the normality of each parameter. A non-parametric test was used when there was no normal data, and a parametric test was used to compare normal data averages, with their respective standard deviations. An *odds ratio (OR)* with a 95% confidence interval was used to evaluate the association with a risk factor, which indicates association if value 1 is not involved. The correlation among the variables was done by Spearman's correlation. Multiple comparisons were done by the Kruskal–Wallis test. All tests were two-tailed and the difference amount was considered to be significant, if it corresponded to *p* < 0.05.

The correlation levels, according to Spearman's factors, were classified as very weak (0.00 to 0.19), weak (0.20 to 0.39), moderate (0.40 to 0.69), strong (0.70 to 0.89), and very strong (0.90 to 1.00)^45^. The *kappa* factor evaluated the concordance between IgE positivity and reported allergies, and it was classified as weak (κ ≤ 0.40), intermediate (0.40 ≤ κ ≤ 0.75), and strong (κ ≥ 0.75)^44^.
